# Genomic characterization of multidrug-resistant extended-spectrum β-lactamase-producing *Vibrio cholerae* O1 strains from 2022 cholera outbreak in Kenya

**DOI:** 10.1093/jac/dkaf224

**Published:** 2025-07-14

**Authors:** Diana Imoli, John M Maingi, Cecilia Mbae, Susan M Kavai, Celestine Wairimu, Sheilla Mundalo, Georgina Odityo, Mary Wairimu, Zelalem Mekuria, Wondwossen Gebreyes, Samuel Kariuki

**Affiliations:** Centre for Microbiology Research, Kenya Medical Research Institute, Nairobi, Kenya; Department of Biochemistry, Microbiology and Biotechnology, Kenyatta University, Nairobi, Kenya; Department of Biochemistry, Microbiology and Biotechnology, Kenyatta University, Nairobi, Kenya; Centre for Microbiology Research, Kenya Medical Research Institute, Nairobi, Kenya; Centre for Microbiology Research, Kenya Medical Research Institute, Nairobi, Kenya; Centre for Microbiology Research, Kenya Medical Research Institute, Nairobi, Kenya; Centre for Microbiology Research, Kenya Medical Research Institute, Nairobi, Kenya; Centre for Microbiology Research, Kenya Medical Research Institute, Nairobi, Kenya; Centre for Microbiology Research, Kenya Medical Research Institute, Nairobi, Kenya; Global One Health Initiative, Ohio State University, Columbus, OH, USA; Global One Health Initiative, Ohio State University, Columbus, OH, USA; Centre for Microbiology Research, Kenya Medical Research Institute, Nairobi, Kenya; Parasites and Microbes Programme, Wellcome Sanger Institute, Cambridge, UK; Drugs for Neglected Disease Initiative- Africa, Nairobi, Kenya

## Abstract

**Background:**

In mid-2021, a global surge in cholera cases was reported. This study characterized *Vibrio cholerae* O1 isolates obtained from faecal samples of cholera-positive cases during the 2022 cholera outbreak in Kenya.

**Methods:**

A total of 202 *V. cholerae* were confirmed through serogroup and serotype characterization by slide agglutination. Susceptibility testing was done using the Kirby–Bauer disc diffusion method, and ESBL production confirmed using the double-disc synergy test. WGS was performed on Illumina and ONT platforms, followed by bioinformatics analysis.

**Results:**

All the isolates were identified as *V. cholerae* O1 of Ogawa serotype, with 99% classified as MDR and 98.5% positive for ESBL production. Notably, the isolates were resistant to azithromycin, one of the recommended antibiotics for cholera treatment. MDR was linked to the acquisition of an *IncC* plasmid (pVCMLK181) carrying seven resistance genes, including *mph*(A), *mph*(E) and *msr*(E), which confer resistance to azithromycin, and the *bla*_PER-7_ ESBL gene. Resistance to nalidixic acid was associated with mutations in QRDRs of *gyrA* and *parC*. The isolates also carried SXT/R391-like ICE, ICE*Vch*Ind5 featuring a 10 kb deletion and mapped to the 7PET-AFR13 lineage. Phylogenetic analysis revealed a close relationship to other highly drug-resistant AFR13 strains reported in Tanzania, Comoros and Mayotte.

**Conclusions:**

The high prevalence of multidrug resistance in cholera isolates emphasizes the need for continuous surveillance to monitor the evolution of MDR *V. cholerae* O1 strains and calls for consideration of deployment of alternative management and prevention options including oral cholera vaccines and long-term improvement of water, sanitation and hygiene (WASH) infrastructure and practice.

## Introduction

Cholera is a severe diarrhoeal illness caused by ingestion of food or water contaminated with faeces of an infected person. Annually, an estimated 2.9 million cases and 95 000 deaths are reported in cholera-endemic areas, with the highest burden observed particularly in sub-Saharan African countries.^1,2^ In Kenya, cholera has been endemic since the first outbreak in 1971, with multiple subsequent waves, making it a priority notifiable disease under Kenya’s Integrated Disease Surveillance and Response strategy.^2–4^ The causative agent, *Vibrio cholerae*, comprises over 200 serogroups, but only serogroups O1 and O139 are associated with epidemic and pandemic cholera.^5^ Since 1817, seven cholera pandemics have been experienced worldwide, with the seventh pandemic, caused by *V. cholerae* O1 biotype El Tor, still ongoing.^6^ Phylogenetic analyses of this pandemic have identified at least three independent but overlapping waves of global transmission in Africa, with 14 introduction events (AFR1, AFR3–15).^7–10^

Antimicrobial resistance (AMR) in *V. cholerae* O1 is attributed to point mutations or horizontal gene transfer facilitated by mobile genetic elements (MGEs) like plasmids of incompatibility type C (*IncC*) and SXT family integrative and conjugative elements (SXT ICE).^11,12^ These MGEs may also facilitate the spread of resistance traits to other pathogenic bacteria, further increasing the burden of AMR.^13^ Recent outbreaks have reported emergence of MDR *V. cholerae* O1 isolates belonging to 7PET-AFR13 sub-lineage. This highly drug-resistant AFR13 strain carrying 14 AMR genes on an *IncC*-type plasmid was first reported in the Zimbabwe 2018 cholera outbreak^14^ and later in Yemen 2018–19^11^ and Lebanon 2022^15^ outbreaks, but these later strains carried a different set of AMR genes on the *IncC* plasmid. The emergence of this MDR *V. cholerae* O1 strain raises significant concerns regarding the effectiveness of standard antibiotic treatment options, and the detection of ESBL strains is particularly concerning, as extended β-lactam antibiotics are often considered a last-resort treatment for severe bacterial infections.

In mid-2021, a global surge in cholera cases occurred, with many countries experiencing elevated high case fatality rates (CFRs) averaging 1.9% worldwide and 2.9% in Africa.^16^ Kenya reported a major outbreak in October 2022,^2^ and by March 2023, 7235 cases with a CFR of 1.6% recorded.^16^ Here, we characterized *V. cholerae* O1 isolated from patients admitted to the cholera treatment centre at Mama Lucy Kibaki Hospital (MLKH), Nairobi, Kenya during the 2022 cholera outbreak. Our findings provide insights into the AMR and genomic characteristics of strains from this outbreak, with the aim of informing public health strategies for prevention and control.

## Materials and methods

### Ethics

This study was conducted in accordance with the Declaration of Helsinki. Ethical approval was obtained from the Scientific and Ethics Review Unit of the Kenya Medical Research Institute (SERU 4905) and a research permit granted by the National Commission for Science, Technology and Innovation (NACOSTI 339440).

### Bacterial isolates

We studied 202 *V. cholerae* isolates that were obtained from faecal samples of patients seeking treatment at MLKH following routine microbiological culture on thiosulfate citrate bile salts (TCBS) medium. Serogroup and serotype were confirmed by slide agglutination using O1-specific polyvalent antisera (Remel, San Diego, CA, USA) and monovalent antisera specific to Inaba and Ogawa serotypes (Deben, Suffolk, UK).

### Antimicrobial susceptibility testing

Antimicrobial susceptibility test was done using the Kirby–Bauer disc diffusion method on Mueller–Hinton agar plates (Oxoid, Basingstoke, UK). The antimicrobials tested (Oxoid) and their corresponding disc concentrations were: ampicillin (10 μg), cefotaxime (30 μg), ceftazidime (30 μg), cefepime (30 μg), imipenem (10 μg), meropenem (10 μg), amoxicillin/clavulanic acid (20/10 μg), doxycycline (30 μg), nalidixic acid (30 μg), ciprofloxacin (5 μg), sulfamethoxazole/trimethoprim (23.75/1.25 μg), chloramphenicol (30 μg), amikacin (30 μg) and azithromycin (15 μg). The diameters of the inhibition zones were measured and interpreted using CLSI M45 interpretive criteria for *Vibrio* species.^17^ For azithromycin, EUCAST interpretive criteria for *Vibrio* species were used^18^ and CLSI M100 interpretative criteria for Enterobacterales used for doxycycline and nalidixic acid.^19^  *Escherichia coli* ATCC 25922 was used to control for growth of bacteria and potency of antibiotic discs.

### Determination of ESBL production

ESBL production was assessed using the double-disc synergy test (DDST).^20^ The antimicrobials tested were ceftazidime and cefotaxime alone, and ceftazidime and cefotaxime in combination with clavulanic acid (BD Biosciences, Franklin Lakes, NJ, USA). ESBL production was indicated by ≥ 5 mm increase in the diameter of the inhibition zone for the cephalosporin/clavulanic acid combination compared with cephalosporin alone.

### WGS

Genomic DNA was extracted from MDR ESBL-producing *V. cholerae* O1 isolates using the Zymo Research Quick-DNA fungal/bacterial DNA kit (Zymo Research, Irvine, CA, USA) according to the manufacturer’s protocol. Library size and concentration were determined using the 4150 TapeStation system (Agilent Technologies, Santa Clara, CA, USA). Limited-cycle PCR was subsequently employed to amplify the tagged DNA and introduce sequencing indexes. To facilitate a limit-of-detection assessment for each sample, we incorporated PhiX Control v3 (Illumina Inc, San Diego, CA, USA) into each sample prior to library preparation. The prepared libraries were loaded onto a reagent cartridge and subjected to clustering on the NextSeq 2000 System. Subsequently, a paired-end sequencing run with 2 × 150 bp reads was executed. The base calls generated by the NextSeq 2000 system were then transformed into FASTQ files. FastQC v.11.7 (Babraham Bioinformatics, Cambridge, UK) was used to evaluate the quality of the sequenced data prior to downstream bioinformatics analysis.

### Long-read sequencing

Isolate MLK181 was selected for long-read sequencing. Genomic DNA was extracted using QIAGEN QIAamp DNA Mini kit (QIAGEN, Hilden, Germany). Libraries for Nanopore sequencing were prepared using NEBNext reagents and the Native Barcoding Kit (SQK-NBD114), then loaded onto an R10.4 FLO-MIN114 flow cell. Sequencing was performed on the MinION platform using MinKNOW v24.06.14. Raw POD5 files were basecalled using Dorado v0.8.1 (https://github.com/nanoporetech/dorado), which also performed barcode and adapter trimming. A hybrid assembly of long and short reads was performed using Unicycler v0.5.1 (https://github.com/rrwick/Unicycler), resulting in two circular chromosomes and one circular plasmid (GenBank accession numbers CP194674–CP194676).

### Genomic data analysis

Short reads were filtered for quality using FastQC v11.7 (Babraham Bioinformatics) and adapter sequences removed using Trimmomatic.^21^ The filtered reads were assembled using SPAdes v4^22^ with the parameters recommended for assembling bacterial isolate genomes. Genome assembly metrics were assessed using QUAST v5.2.0.^23^ GTDB-Tk v2.4.0 was used to taxonomically classify the genomes and confirm that the sequenced reads originated from *V. cholerae* and not contaminants.^24^ AMR genes and resistance-associated point mutations were identified using AMRFinderPlus v3.11.^25^ SXT ICE was identified by searching for sequences similar to ICE*Vch*Ind5 (GenBank: GQ463142.1) using BLAST+ 2.15.0 on proksee.ca/ and plasmid visualized using BRIG v0.95.^26^

### Genomic classification and phylogenetic analysis

The assembled genomes confirmed as *V. cholerae* were uploaded to the Cholera Finder v1.0 database on the Center for Genomic Epidemiology (CGE) (CGE Server (dtu.dk) for classification based on serogroup, biotype and wave, and subsequently uploaded to Pathogenwatch (https://pathogenwatch) to determine the ST and sub-lineage. In addition, 15 genomes of previously reported sub-lineages in Kenya and 43 global AFR13 strains’ genomes described by Rouard^27^ were included in the phylogenetic analysis (Table S1, available as Supplementary data at *JAC*  Online). Parsnp v2.1.3 was used to extract SNPs and perform alignment using *V. cholerae* O1 El Tor N16961 as the reference. A maximum-likelihood phylogenetic tree was then constructed using IQ-TREE2 v2.4.0^28^ with 1000 bootstraps and visualized with iTOL v7 (itol.embl.de/).

## Results

### Characteristics and distribution of cholera cases during the 2022 outbreak

**Figure 1.**
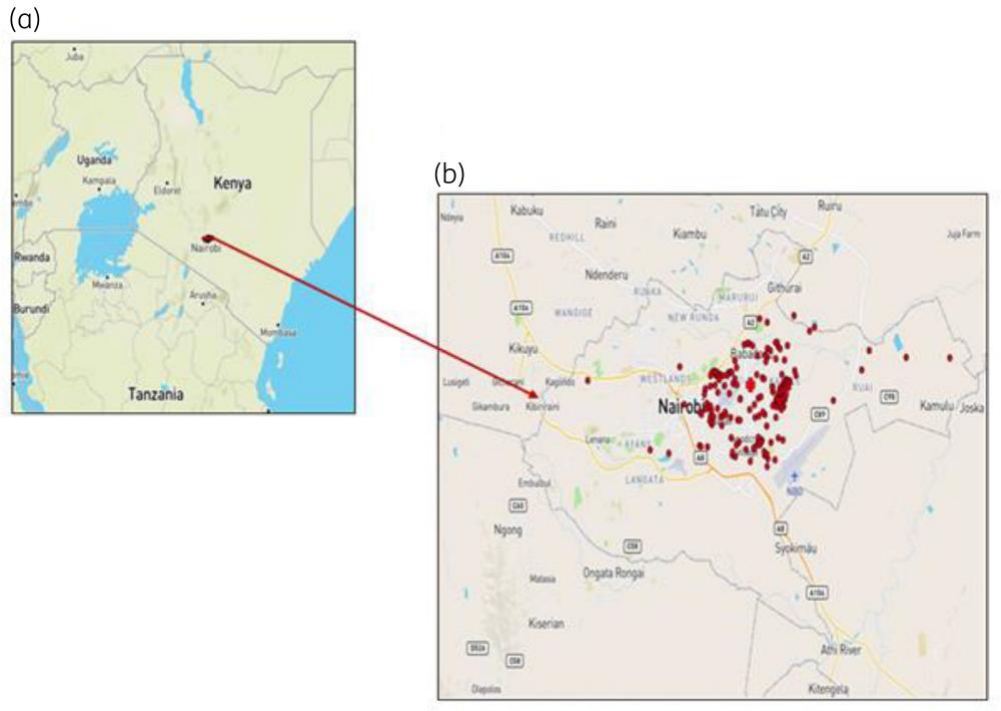
Map showing the geographical distribution of the 202 cholera cases from the 2022 cholera outbreak. (a) Map of Kenya showing the location of Nairobi. (b) Map of Nairobi showing distribution of the cases within the areas in Nairobi. The maroon dots indicate the areas from which the cases originated, while the red cross marks the location of MLKH.

All 202 isolates were *V. cholerae* O1 of Ogawa serotype. The majority of the isolates were from males (65.3%). The age of the patients ranged from 1 to 63 years, with 24–45 years being the most affected age group (51.5%) (Table 1). All the cases were within the Nairobi region, with the majority originating from areas surrounding MLKH (Figure 1).

**Table 1.** Demographic characteristics of cholera-positive patients at MLKH during the 2022 outbreak

Characteristic	*n* (*N* = 202)	%
Gender		
Female	70	34.7
Male	132	65.3
Age group (years)		
1–14	24	11.9
15–24	40	19.8
25–44	104	51.5
45–63	34	16.8

### Antimicrobial susceptibility patterns

**Figure 2.**
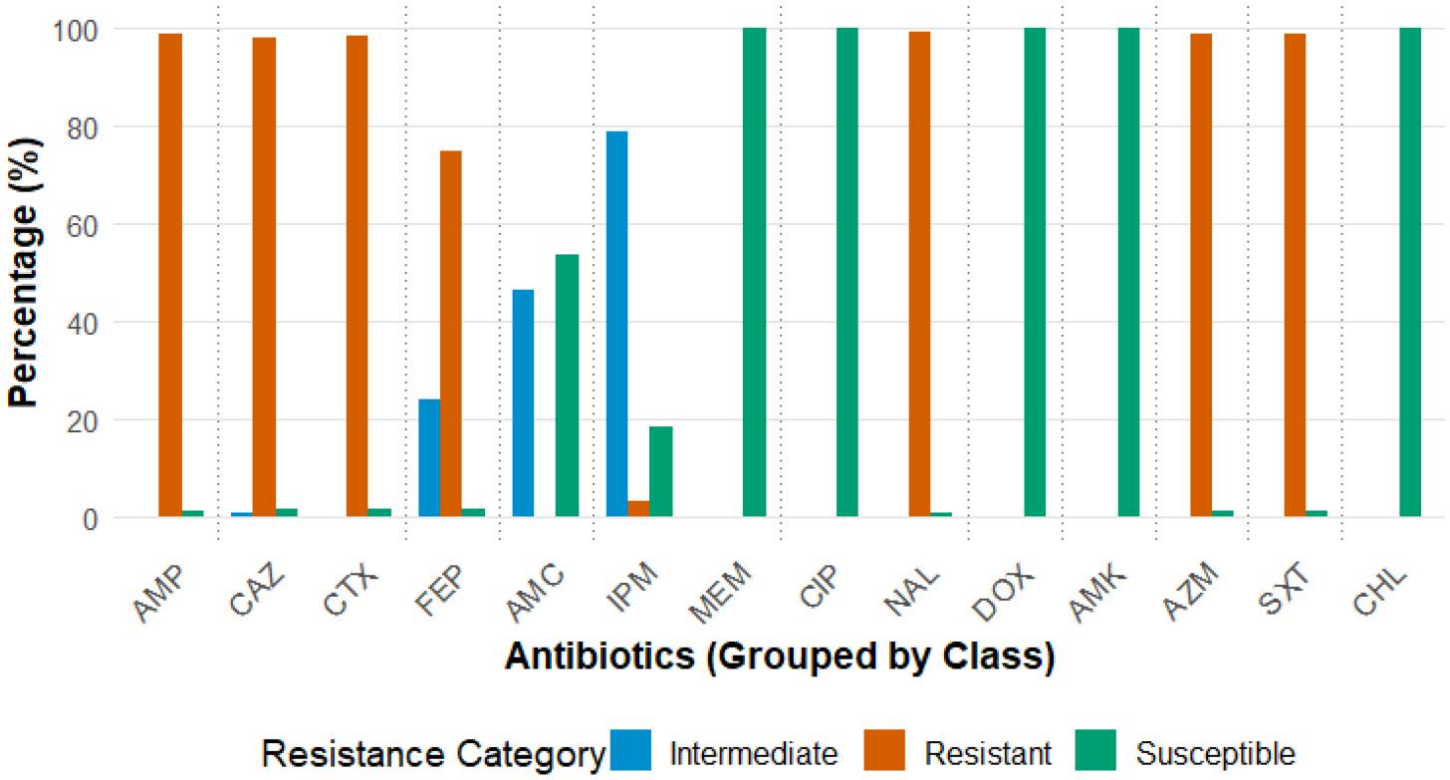
Proportions of antimicrobial susceptibility testing patterns for *V. cholerae* O1 isolates from the 2022 cholera outbreak in Kenya. The dashed horizontal line indicates the different antibiotic classes. AMP, ampicillin; CAZ, ceftazidime; CTX, cefotaxime; FEP, cefepime; AMC, amoxicillin/clavulanic acid; IPM, imipenem; MEM, meropenem; CIP, ciprofloxacin; NA, nalidixic acid; DOX, doxycycline; AMK, amikacin; AZM, azithromycin; CHL, chloramphenicol; SXT, trimethoprim/sulfamethoxazole.

Of the 202 *V. cholerae* O1 isolates tested, 200 (99%) were classified as MDR^29^ and 199 (98.5%) were positive for ESBL production. The MDR isolates showed resistance to up to 8 antimicrobial classes out of the 11 classes tested. Only one isolate was susceptible to all the antimicrobial classes tested. A high prevalence of resistance to third- and fourth-generation cephalosporins was observed, with 199/202 (98.5%) resistant to cefotaxime, 198/202 (98%) resistant to ceftazidime and 151/202 (74.7%) resistant to cefepime (Figure 2). Additionally, 99% (200/202) showed resistance to ampicillin, azithromycin and trimethoprim/sulfamethoxazole, while 99.5% (201/202) were resistant to nalidixic acid. Reduced susceptibility was observed to imipenem (159/202; 78.7%), a carbapenem, and amoxicillin/clavulanic acid (94/202; 46.5%), a β-lactam/β-lactamase inhibitor combination. All the isolates were susceptible to doxycycline, chloramphenicol, amikacin, ciprofloxacin and meropenem.

### AMR determinants

All 41 MDR and ESBL-producing isolates exhibited resistance genes that corresponded with their phenotypic resistance profiles (Table 2). *mph*(A), *msr*(E) and *mph*(E) genes were linked to azithromycin resistance, while *bla*_PER-7_ was linked to resistance to β-lactams. All the sequenced isolates harboured QRDR mutations in the DNA gyrase gene (*gyrA*) and topoisomerase IV gene (*parC*), resulting in amino acid substitutions Ser83Ile in *gyrA* and Ser85Leu in *parC*. These mutations were linked to resistance to nalidixic acid.

**Table 2.** Distribution of associated AMR genes in the 41 sequenced *V. cholerae* O1 isolates from the 2022 cholera outbreak in Kenya

Phenotypic resistance profile	Antibiotic class	Resistance genes (% isolates)
AMP, CAZ, CTX, FEP, AMC	β-Lactams	*bla*_PER-7_ (100)
	Aminoglycoside	*aadA2* (100)
SXT	Sulphonamides	*sul1* (100)
	Trimethoprim	*dfrA1* (100)
AZM	Macrolides	*mph*(A) (100)
		*msr*(E) (100)
		*mph*(E) (100)
NA	QRDR mutations	*gyrA_*S83I (100)
		*parC_*S85L (100)

AMP, ampicillin; CAZ, ceftazidime; CTX, cefotaxime; FEP, cefepime; AMC, amoxicillin/clavulanic acid; SXT, trimethoprim/sulfamethoxazole; AZM, azithromycin; NA, nalidixic acid

### AMR gene-containing elements

**Figure 3.**
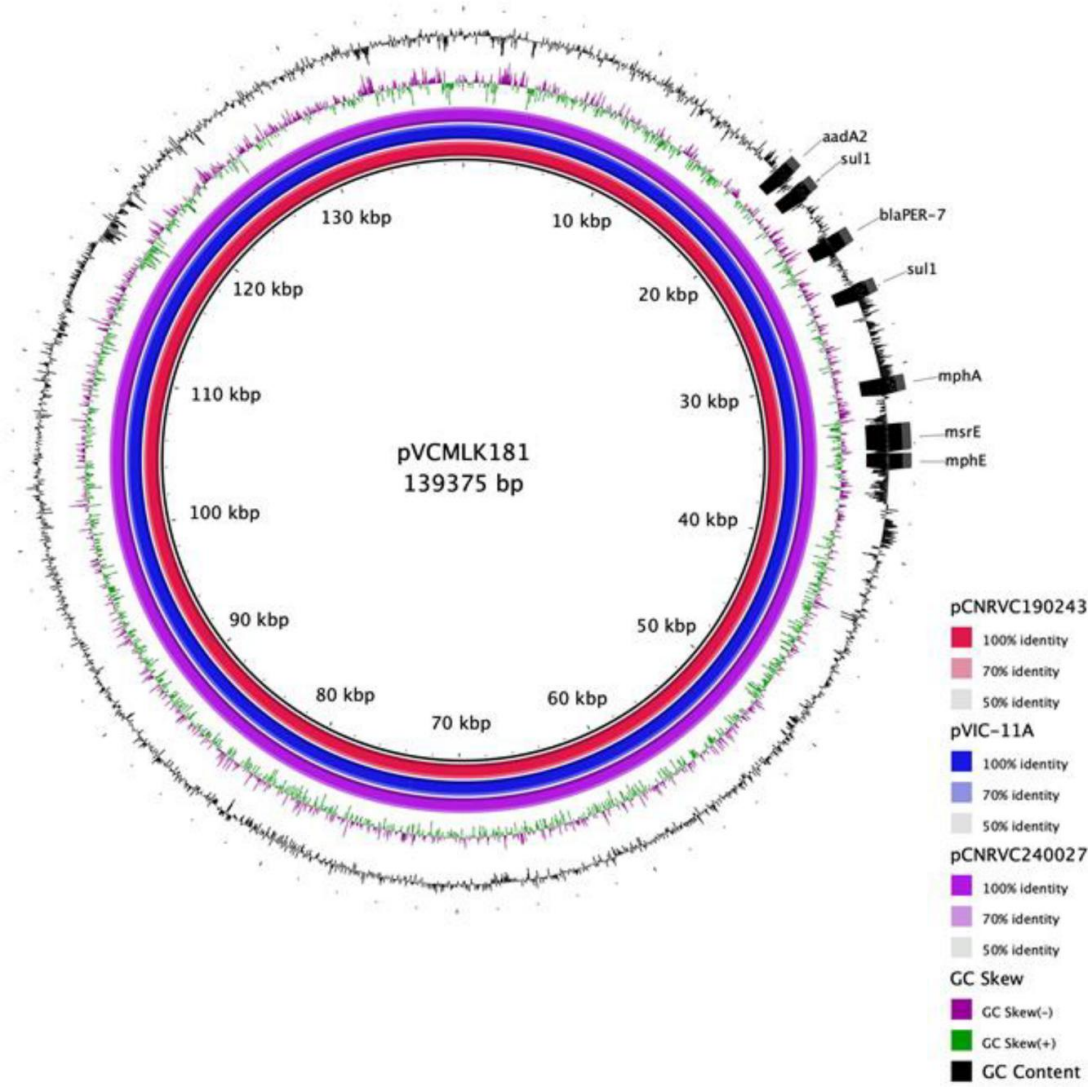
Comparative analysis of MDR *IncC* plasmids from Kenya (GenBank accession no CP194676), Yemen (GenBank accession no. OW443149), Lebanon (GenBank accession no. CP134059.1) and Mayotte (GenBank accession no. CP158976). The innermost circle represents the nuclear positions of plasmid pVCMLK181 (Kenya) with annotated AMR genes. The red, blue and purple rings represent regions of pCNRVC190243 (Yemen), pVIC-11A (Lebanon) and pCNRVC240027 (Mayotte), respectively, showing 100% nucleotide sequence identity to plasmid pVCMLK181.

All sequenced isolates carried an *IncC* plasmid (pVCMLK181) of 139 kb, which contained *sul1*, *bla*_PER-7_, *aadA2*, *mph*(A), *msr*(E) and *mph*(E) resistance genes (Figure 3). pVCMLK181 displayed 100% nucleotide sequence identity to pCNRVC190243, pVIC-11A and pCNRVC240027, which were previously identified in highly drug-resistant AFR13 strains from Yemen (2018–19),^11^ Lebanon (2022–23)^15^ and Mayotte (2023)^27^ outbreaks. Additionally, all isolates carried the SXT/R391-like ICE, ICE*Vch*lnd5, which featured a deletion of approximately 10 kb resulting in the loss of resistance genes to chloramphenicol (*floR*), sulphonamides (*sul2*) and aminoglycosides [*aph(3″)-Ib*, *aph(6)-Id*] but retaining the *dfrA1* gene, which confers resistance to trimethoprim.

### Genomic characterization and phylogenetic relatedness

All the sequenced isolates were classified as *V. cholerae* O1 of El Tor Variant biotype (ST69) and fell within Late Wave 3 of the ongoing seventh pandemic and were contained in the 13-transmission event (AFR13) (Table 3).

**Table 3.** Genome characteristics of the MDR strains from the 2022 Kenya cholera outbreak

Species	*V. cholerae*
Serogroup, serotype and biotype	O1, Ogawa, El Tor variant
Genomic lineage and sub-lineage	Seventh pandemic, AFR13
ST and wave	ST69, Late Wave 3
Genetic markers	*ompW*, *rfbV_O1*, *rstR*_*et*, *tcpA*_*et3*, *ctxB*_*7*, *VC*2346

**Figure 4.**
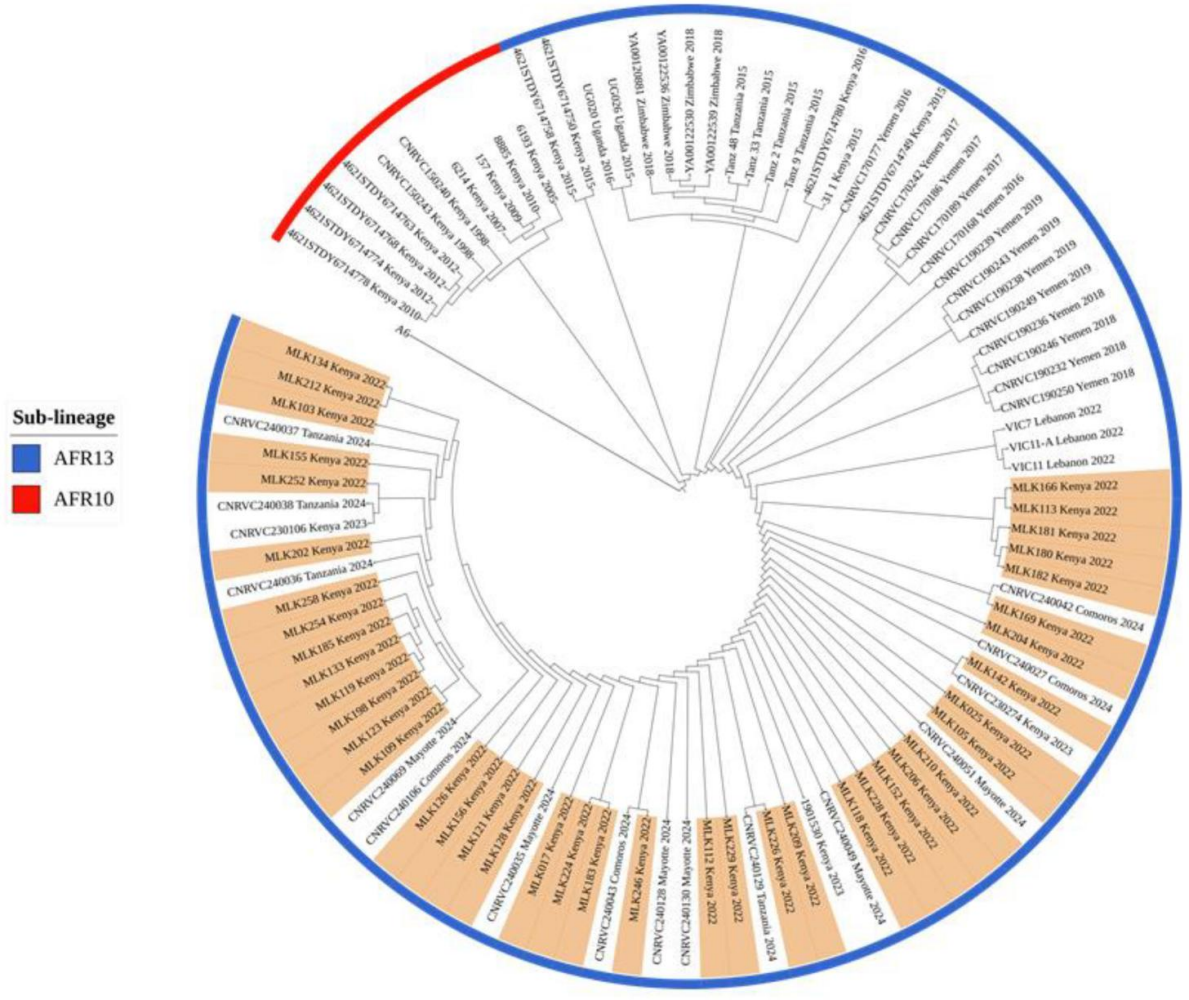
Maximum-likelihood phylogenetic tree of 100 *V. cholerae* O1 AFR13 strains and previously reported AFR10 Kenyan strains. The tree is constructed based on SNP differences and rooted using the A6 genome, the most ancestral 7PET strain. Kenyan AFR13 strains from the 2022 cholera outbreak are highlighted in colour.

Phylogenetic analysis showed the 2022 Kenya outbreak isolates to be more genetically similar as they clustered closely on the same branches with minimal genetic divergence, as indicated by the few SNPs (Figure 4; Table S2). The isolates clustered closely with AFR13 strains that were isolated in Mayotte from three European travellers with a history of travel to Kenya in 2023,^27^ and also showed close relatedness to other circulating drug-resistant strains reported in Tanzania and Comoros.

## Discussion

All 202 isolates analysed in this study belonged to the Ogawa serotype, consistent with the 2015–16 cholera outbreak in Kenya^30^ but in contrast with earlier outbreaks in 2005–07, 2007–10 and 2014, where serotype Inaba was predominant.^31,32^ This observed switch between Inaba and Ogawa is common in cholera-endemic settings.^33^

Oral rehydration is the primary treatment for cholera but doxycycline, azithromycin and ciprofloxacin are recommended as first-line antibiotics for patients with severe disease to shorten the duration of diarrhoea and limit bacterial shedding.^34,35^ In a previous study, Njeru *et al.*^32^ reported that isolates from the 2007–10 outbreak in Kenya were susceptible to azithromycin, contrasting with our findings, where 99% of the isolates were resistant to azithromycin. This suggests that azithromycin may no longer be effective for cholera treatment in Kenya. However, all isolates in this study were susceptible to doxycycline, indicating that doxycycline remains an effective option for cholera management.

The MDR phenotype in our study is attributed to the acquisition of a 139 kb *IncC* plasmid (pVCMLK181) carrying seven resistance genes, with a backbone similar to pCNRVC190243, pVIC-11A and pCNRVC240027 MDR plasmids identified in Yemen, Lebanon and Mayotte. The isolates also carry SXT/R391 ICE (ICE*Vch*Ind5) with an approximately 10 kb deletion. The presence of SXT ICE has been suggested to prevent stable replication of *IncC* plasmid through an unknown functional interference.^11^ It is thought that this deletion, which has been observed in 7PET Wave 3 strains, could potentially disrupt this interference, enabling the coexistence and stable replication of both SXT ICE and *IncC* plasmid, a characteristic seen in these highly drug-resistant AFR13 outbreak strains.^11,15,27^

Previously, ESBL-positive *V. cholerae* O1 strains were rarely reported and only associated with sporadic cases and minor outbreaks.^36^ In Kenya, Weill *et al.*^37^ reported three AFR10 strains carrying the *bla*_CTX-M-15_ ESBL gene during 2010 and 2012 outbreaks. Kariuki *et al.*^38^ also reported strains belonging to ST69, which carried the *bla*_CTX-M-15_ ESBL gene on an *IncC* plasmid (pVC_ESBL). In this study, we found a high prevalence of resistance to β-lactam antibiotics, with plasmid-borne *bla*_PER-7_ detected as the ESBL-encoding gene. These AFR13 strains carrying the *bla*_PER-7_ ESBL gene is consistent with the strains reported in outbreaks in Yemen in 2019,^11^ Lebanon in 2022^15^ and Mayotte in 2023.^27^ However, this contrasts with the Zimbabwe 2019 outbreak, where ESBL-producing AFR13 strains were found to carry the *bla*_CTX-M-15_ gene on a 160 kb *IncC* plasmid, pYA00120881, which had a different MDR region compared with plasmid pVCMLK181.^14^

All sequenced isolates had QRDR mutations, Ser83Ile in *gyrA* and Ser85Leu in *parC* genes, which corroborated the nalidixic acid resistance. However, these mutations were not associated with ciprofloxacin resistance, as the isolates remained phenotypically susceptible to ciprofloxacin based on both CLSI M45 and EUCAST interpretation criteria for *Vibrio* species. Recent studies have reported these highly resistant AFR13 strains to also show reduced susceptibility to ciprofloxacin, although using MIC values,^15,27,37^ as opposed to our study, which used inhibition zone measurements. Our findings are consistent with those of Hounmanou and colleagues,^39^ who also used data on zones of inhibition and reported QRDR mutations without phenotypic ciprofloxacin resistance. All the sequenced isolates carried the *catB9* resistance gene yet remained phenotypically susceptible to chloramphenicol. This discrepancy between phenotypic and genotypic resistance profiles has been observed in other studies,^39,40^ and the presence of the *cat*B9 gene has not previously been associated with resistance to chloramphenicol. The isolates also harboured the *varG* carbapenem gene; while *varG* has not yet been accepted as a resistance gene, 78% of the isolates exhibited reduced susceptibility to imipenem, although all remained susceptible to meropenem.

All the sequenced isolates belonged to 7PET ST69 and the AFR13 sub-lineage, which was introduced to East Africa from South Asia, and have since been responsible for recurring cholera outbreaks in the region.^39,41^ Previously, AFR13 strains were susceptible to most commonly available antibiotics, but recent outbreaks have shown a shift toward highly drug-resistant variants. The close genetic relatedness of Kenya 2022 strains with those from Comoros, Tanzania and Mayotte indicates that a common strain may be responsible for these outbreaks, supporting evidence of regional cholera spread and highlighting the role of human-mediated transmission around the globe.^11,15,27^

The one pan-susceptible isolate in this study is in contrast with the resistance trends observed in 7PET ST69/ST515 as none of these pandemic strains have been pan-susceptible since the 2000s. This pan-susceptible isolate was not sequenced and perhaps could be of other STs like ST75. Toxigenic *V. cholerae* O1 of ST75 and lacking *Vibrio* seventh pandemic island I (VSP-I) and VSP-II have recently been isolated from both clinical cases and environmental samples in South Africa,^42^ and in some regions like Taiwan, ST75 strains have replaced the predominant ST69.^43^

This study faced limitations. First, the samples analysed were obtained exclusively from one healthcare facility, which means that most patients were from the surrounding area and therefore it is not feasible to generalize for all the informal settlement. Second, we were only able to sequence a small proportion of the isolates. As a result, we cannot rule out the possibility that other *V. cholerae* strain*s* may be present in the region. Third, the study did not collect behavioural data from patients, which constrains the identification of potential risk factors associated with the cholera outbreak.

In conclusion, the emergence of MDR and ESBL-producing 7PET-AFR13 strains that carry the *IncC* plasmid poses a challenge for management of cholera. If these strains spread to other geographic locations or transfer their plasmid to other strains or bacterial pathogens, it could further complicate treatment efforts. Continuous surveillance is needed to monitor the evolution of MDR *V. cholerae* O1 strains in endemic settings. In addition, deployment of control and prevention strategies, including oral cholera vaccines in the short term and improvement of water, sanitation and hygiene (WASH) infrastructure and practices in the long term, would benefit this vulnerable population.

## Supplementary Material

dkaf224_Supplementary_Data

## Data Availability

This Whole Genome Shotgun project has been deposited at DDBJ/ENA/GenBank under BioProject PRJNA1186719.

## Supplementary data

Tables S1 and S2 are available as Supplementary data at *JAC*  Online.
