# Supplementary material for: Genomic characterization of multidrug-resistant extended-spectrum β-lactamase-producing Vibrio cholerae O1 strains from 2022 cholera outbreak in Kenya
Source: J Antimicrob Chemother. 2025 Jul 14;80(9):2399–407. doi: 10.1093/jac/dkaf224 (PMC12404722; doi:10.1093/jac/dkaf224)
Supplement: dkaf224_Supplementary_Data [file dkaf224_supplementary_data.zip › Table S1.docx]

| **Accession numbers for the additional genomes in phylogenetic analysis** | | | | |
| --- | --- | --- | --- | --- |
| **Isolate ID** | **Country** | **Year** | **Accession number** | **Source** |
| CNRVC230274 | Kenya | 2023 | ERS20275829 | Rouard et al. N Engl J Med 2020 |
| H231240238_1901530 | Kenya | 2023 | SRR26217611 | Rouard et al. N Engl J Med 2020 |
| CNRVC230106 | Kenya | 2023 | ERS20275828 | Rouard et al. N Engl J Med 2020 |
| CNRVC240128 | Mayotte | 2024 | ERS20275841 | Rouard et al. N Engl J Med 2020 |
| CNRVC240042 | Comoros | 2024 | ERS20275835 | Rouard et al. N Engl J Med 2020 |
| CNRVC240051 | Mayotte | 2024 | ERS20275838 | Rouard et al. N Engl J Med 2020 |
| CNRVC240027 | Comoros | 2024 | ERS20275830 | Rouard et al. N Engl J Med 2020 |
| CNRVC240037 | Tanzania | 2024 | ERS20275833 | Rouard et al. N Engl J Med 2020 |
| CNRVC240130 | Mayotte | 2024 | ERS20275843 | Rouard et al. N Engl J Med 2020 |
| CNRVC240106 | Comoros | 2024 | ERS20275840 | Rouard et al. N Engl J Med 2020 |
| CNRVC240043 | Comoros | 2024 | ERS20275836 | Rouard et al. N Engl J Med 2020 |
| CNRVC240038 | Tanzania | 2024 | ERS20275834 | Rouard et al. N Engl J Med 2020 |
| CNRVC240036 | Tanzania | 2024 | ERS20275832 | Rouard et al. N Engl J Med 2020 |
| CNRVC240069 | Mayotte | 2024 | ERS20275839 | Rouard et al. N Engl J Med 2020 |
| CNRVC240035 | Comoros | 2024 | ERS20275831 | Rouard et al. N Engl J Med 2020 |
| CNRVC240049 | Mayotte | 2024 | ERS20275837 | Rouard et al. N Engl J Med 2020 |
| CNRVC240129 | Tanzania | 2024 | ERS20275842 | Rouard et al. N Engl J Med 2020 |
| 31_1 | Kenya | 2015 | ERR2265589 | Weill et al. Nature 2019 |
| 4621STDY6714780 | Kenya | 2016 | ERS1572815 | Weill et al. Nature 2019 |
| 4621STDY6714758 | Kenya | 2015 | ERS1572793 | Weill et al. Nature 2019 |
| 4621STDY6714750 | Kenya | 2015 | ERS1572785 | Weill et al. Nature 2019 |
| 4621STDY6714749 | Kenya | 2015 | ERS1572784 | Weill et al. Nature 2019 |
| UG026 | Uganda | 2015 | SAMN08744332 | Bwire et al. PLoS Negl Trop Dis 2018 |
| UG020 | Uganda | 2016 | SAMN08744331 | Bwire et al. PLoS Negl Trop Dis 2018 |
| 4621STDY6714774 | Kenya | 2012 | ERS1572809 | Weill et al. Nature 2019 |
| 4621STDY6714778 | Kenya | 2010 | ERS1572813 | Weill et al. Nature 2019 |
| 4621STDY6714768 | Kenya | 2012 | ERS1572803 | Weill et al. Nature 2019 |
| 4621STDY6714763 | Kenya | 2012 | ERS1572798 | Weill et al. Nature 2019 |
| CNRVC150243 | Kenya | 1998 | ERR1879540 | Weill et al. Science 2017 |
| CNRVC150240 | Kenya | 1998 | ERR1879539 | Weill et al. Science 2017 |
| 6193 | Kenya | 2005 | ERR019296 | Mutreja et al. Nature 2011 |
| 6214 | Kenya | 2007 | ERR019287 | Mutreja et al. Nature 2011 |
| 8885 | Kenya | 2010 | ERR117595 | Kiiru et al. Plos One 2013 |
| 157 | Kenya | 2009 | ERR117592 | Kiiru et al. Plos One 2013 |
| Tanz_9 | Tanzania | 2015 | ERS2318682 | Kachwamba et al. BMC Infect Dis 2017 |
| Tanz_2 | Tanzania | 2015 | ERS2318680 | Kachwamba et al. BMC Infect Dis 2017 |
| Tanz_48 | Tanzania | 2015 | ERS2318700 | Kachwamba et al. BMC Infect Dis 2017 |
| Tanz_33 | Tanzania | 2015 | ERS2318693 | Kachwamba et al. BMC Infect Dis 2017 |
| YA00122530 | Zimbabwe | 2018 | ERR3342507 | Mashe et al. N Engl J Med 2020 |
| YA00122536 | Zimbabwe | 2018 | ERR3342512 | Mashe et al. N Engl J Med 2020 |
| YA00120881 | Zimbabwe | 2018 | ERR3342506 | Mashe et al. N Engl J Med 2020 |
| YA00122539 | Zimbabwe | 2018 | ERR3342514 | Mashe et al. N Engl J Med 2020 |
| VIC11 | Lebanon | 2022 | ERS16287304 | Abou Fayad et al. bioRxiv 2023 |
| VIC7 | Lebanon | 2022 | ERS16287318 | Abou Fayad et al. bioRxiv 2023 |
| VIC11-A | Lebanon | 2022 | ERS16287303 | Abou Fayad et al. bioRxiv 2023 |
| CNRVC170177 | Yemen | 2016 | ERR2269615 | Weill et al. Nature 2019 |
| CNRVC170168 | Yemen | 2016 | ERR2265674 | Weill et al. Nature 2019 |
| CNRVC170189 | Yemen | 2017 | ERR2269644 | Weill et al. Nature 2019 |
| CNRVC170186 | Yemen | 2017 | ERR2269641 | Weill et al. Nature 2019 |
| CNRVC170242 | Yemen | 2017 | ERR2269834 | Weill et al. Nature 2019 |
| CNRVC190238 | Yemen | 2019 | ERR7057858 | Lassalle et al. Nat Microbiol 2023 |
| CNRVC190249 | Yemen | 2019 | ERR7057899 | Lassalle et al. Nat Microbiol 2023 |
| CNRVC190243 | Yemen | 2019 | ERR7057893 | Lassalle et al. Nat Microbiol 2023 |
| CNRVC190239 | Yemen | 2019 | ERR7057859 | Lassalle et al. Nat Microbiol 2023 |
| CNRVC190232 | Yemen | 2018 | ERR7057835 | Lassalle et al. Nat Microbiol 2023 |
| CNRVC190250 | Yemen | 2018 | ERR7057900 | Lassalle et al. Nat Microbiol 2023 |
| CNRVC190236 | Yemen | 2018 | ERR7057856 | Lassalle et al. Nat Microbiol 2023 |
| CNRVC190246 | Yemen | 2018 | ERR7057896 | Lassalle et al. Nat Microbiol 2023 |
| A6 | Indonesia | 1958 | ERR025382 | Mutreja et al. Nature 2011 |
